# From uniform to belonging: how fashion imagery of school uniforms shapes students’ psychological capital via social identification

**DOI:** 10.3389/fpsyg.2026.1773948

**Published:** 2026-02-24

**Authors:** Yifan Di, Lixian Liu, Yudian Zhang

**Affiliations:** 1 School of Fashion Design and Engineering, Zhejiang Sci-Tech University, Hangzhou, Zhejiang, China; 2 Silk and Fashion Culture Research Center of Zhejiang Province, Zhejiang Sci-Tech University, Hangzhou, Zhejiang, China; 3 School of Art and Design, Zhejiang Sci-Tech University, Hangzhou, Zhejiang, China

**Keywords:** fashion imagery, PLS-SEM, psychological capital, school uniform, social identification

## Abstract

**Introduction:**

School uniforms play a crucial role in students’ psychological development; however, existing designs often neglect students’ individualized aesthetic needs, potentially hindering their mental health and emotional growth. This study explores whether students’ perceptions of the fashion imagery of school uniforms can act as an educational contextual cue that enhances their psychological capital through group identification.

**Methods:**

Expert-reviewed and standardized uniform images were used as visual stimuli, with 210 Chinese participants completing a structured questionnaire. Principal component analysis (PCA) identified three aesthetic orientations—individuality-oriented, responsibility-oriented, and function-oriented aesthetics—while partial least squares structural equation modeling (PLSSEM) was employed to test both direct and indirect pathways among variables.

**Results:**

The results indicate that function-oriented aesthetics show a clear positive association with psychological capital, whereas responsibility-oriented aesthetics are positively related to psychological capital primarily through group identification, despite their direct effect being marginally significant. In contrast, the association between individuality-oriented aesthetics and group identification was not significant, suggesting a distinct, self-focused psychological pathway. The model explained *R*^2^ = 0.260 for social identification and *R*^2^ = 0.415 for psychological capital.

**Discussion:**

This study provides empirical evidence for the pivotal role of visual cues in school uniform design—such as inclusivity, sustainability, comfort, and safety—in fostering students’ sense of belonging and the development of positive psychological resources, offering both theoretical and practical insights for school uniform design and campus culture development from a psychological perspective.

## 1 Introduction

Global transformations in education are shifting from a teacher-centered paradigm toward a learner-centered governance framework (Briffett-Aktaş and Ying, 2025; Dewey, 1986). This transition not only requires innovation in curricula and pedagogy but also calls for a comprehensive re-examination of every element within educational environments (Knox, 2022; Makaremi et al., 2024), along with increased attention to students’ psychological and socio-emotional development (Hammill et al., 2022; He et al., 2024; Woof et al., 2021). Within this process, school uniforms—highly institutionalized and standardized attire encountered daily by students—are being reinterpreted as important cultural carriers of educational experience and student development (Friedrich and Shanks, 2023). School uniforms influence not only physical comfort and usability but also play a significant role in psychological development and socialization (Ansari et al., 2022; Friedrich and Shanks, 2023). However, current uniform design and decision-making often prioritize institutional regulations over students’ aesthetic and psychological experiences. This neglect is particularly pronounced during adolescence—a critical period for the awakening of aesthetic awareness and the formation of self-identity—when students exhibit strong sensitivity and personal preferences regarding clothing styles, colors, and coordination (Beaudoin and Lachance, 2006; Piacentini and Mailer, 2004). As fashion increasingly becomes a medium of self-expression among youth, the appearance of school uniforms has acquired symbolic meaning. When the standardized uniform system conflicts with individual aesthetic needs, students’ perceptions of the fashion imagery of uniforms may become a key variable influencing their emotional experience and psychological development.

Existing research has extensively explored the social functions of school uniforms in promoting discipline, equality, and school cohesion (Ansari et al., 2022; Woo et al., 2020), as well as their role in shaping students’ identity and sense of collective belonging (Sabic-El-Rayess et al., 2020). However, such studies often view school uniforms as homogeneous, static institutional symbols and give limited attention to students’ subjective aesthetic perceptions of school uniforms and their potential psychological consequences. Given that school uniforms serve as a form of standardized attire, their core social function lies in shaping the identity of student groups. Social Identity Theory (SIT) provides a crucial theoretical framework for understanding this process. According to this theory, individuals define themselves through their group membership (Tajfel and Turner, 2001). From this perspective, when students perceive that a school uniform reflects both group values and aligns with their aesthetic expectations, the fashion imagery of the uniform can become a positive group symbol, thereby strengthening their sense of belonging to the school community and facilitating psychological development.

Social identity is not only a social emotional bond but also a social-psychological resource that fosters individual development. Research has shown that social identity can enhance an individual’s sense of wellbeing, self-efficacy, and psychological resilience (Camus et al., 2024; Greenaway et al., 2015). These positive experiences are closely aligned with the concept of “psychological capital” in positive psychology. Psychological capital is defined as a state of positive psychological development, comprising self-efficacy, hope, resilience, and optimism, and has been proven to effectively promote learning engagement and mental health (Edalat et al., 2025; Luthans et al., 2006; Tho and Quynh Thu, 2026). Therefore, social identity can be viewed as an important social-psychological foundation for the formation of psychological capital.

Based on this, the current study has two main objectives: (1) to extract the core aesthetic dimensions of students’ perceptions of school uniform fashion imagery; and (2) to examine the direct and indirect effects of different aesthetic types on psychological capital, with a focus on the mediating role of social identity. To achieve these objectives, the study employed expert review methods and standardized school uniform images as visual stimuli and administered a questionnaire survey to participants with firsthand experience of wearing school uniforms during their student years. Using principal component analysis (PCA), three core aesthetic orientations were identified: individual-oriented aesthetics, responsibility-oriented aesthetics, and function-oriented aesthetics. Subsequently, Partial Least Squares Structural Equation Modeling (PLS-SEM) was used to further examine the relationships between the variables. Theoretically, this study shifts the focus of school uniform research from institutional norms to students’ psychological development, revealing the potential mechanism through which aesthetic perception promotes positive psychological growth via social identification. From a practical perspective, the findings provide empirical support for educators and school uniform designers, emphasizing the need to consider students’ subjective perceptions of aesthetics and collective values in uniform design and campus culture development, thereby enhancing students’ sense of school belonging and promoting the formation of psychological capital.

## 2 Literature review

### 2.1 Fashion imagery

The concept of Kansei imagery originates from Japanese Kansei Engineering, which focuses on the subjective emotions and aesthetic impressions evoked when individuals perceive a product’s appearance, texture, and form (Nagamachi, 1995) The core of this theory lies in the interaction between emotion and cognition during perception, providing a psychological foundation for understanding how people construct emotional meaning through visual stimuli (Norman, 2007). From a perceptual psychology perspective, such imagery can be understood as a set of aesthetic perceptual cues or symbolic affordances embedded in visual form, through which individuals interpret social meaning and identity-related information (Alton et al., 2025; Norman, 2007). Accordingly, this study introduces the concept of fashion imagery into clothing design, exploring how visual design bridges aesthetic psychology and social identity. Clothing functions not only as a physical object but also as a social symbol that conveys cultural value and identity through form, color, and material.

Compared to general clothing, school uniforms, as normative symbols within the educational system, serve a dual function in socialization and identity construction (Stanley, 1996). In the highly socialized environment of schools, students’ aesthetic perceptions of school uniforms are not only related to aesthetic preferences but also reflect their sense of position within collective culture and psychological identity. In adolescent groups, clothing serves as a medium for self-expression and social belonging, as well as a practice for demonstrating aesthetic ability and cultural differentiation (Badaoui et al., 2018; Jenkinson, 2020). This perceptual process can be understood as “aesthetic socialization,” where individuals gradually internalize social values and reconstruct personal aesthetic standards and cultural identities through the imitation and reprocessing of popular styles and aesthetic norms (Bandura, 1997; Dagalp and Hartmann, 2022).

With the shift in educational philosophies and the increasing agency of students, school uniforms have gradually transitioned from institutional symbols to cultural symbols (Friedrich and Shanks, 2023; Wolfe, 2024). Adolescents increasingly view clothing as a vehicle for self-expression and social identity, making the fashion design of school uniforms an important means for individual expression and group integration. Students’ aesthetic evaluations of school uniforms involve not only visual appeal but also psychological judgments about social identity and a sense of belonging (Hester and Hehman, 2023). When school uniforms feature fashion elements, students are more likely to integrate the school symbol with their personal image, thus enhancing their level of social identity.

From a social-psychological perspective, the process of students perceiving the fashion imagery of school uniforms reflects the interaction between aesthetic needs and social identity. While pursuing self-expression, individuals also seek belonging and identity affirmation through collective symbols (Abrams, 2009; Worley and Smith, 2026). This interaction helps students achieve a psychological balance between individual aesthetic demands and institutional norms, promoting positive identification with the school community. In this sense, fashion imagery is not merely a visual stimulus, but an environmental psychological cue that carries symbolic information and affords social meaning construction. Therefore, the fashion imagery of school uniforms not only acts as an external symbol of school culture but also serves as a psychological trigger for generating students’ social identity and positive psychological resources. Based on this, the present study views school uniform fashion imagery as a key psychological link connecting educational aesthetics with students’ psychological development.

### 2.2 Social identification

Social Identity Theory (SIT), proposed by Henri Tajfel and John Turner, emphasizes the role of group membership in defining the self. According to this theory, an individual’s self-concept is shaped not only by personal traits and experiences but also by their group membership. By categorizing themselves as members of specific groups (such as school, ethnicity, or culture), individuals gain a sense of belonging, self-esteem, and emotional support. The formation of social identity is often accompanied by a process of social comparison, where individuals enhance their identification with and loyalty to the in-group by contrasting it with out-groups (Tajfel and Turner, 2001).

In the educational context, schools serve not only as institutions for knowledge transfer but also as critical environments for learning social identity and internalizing values (Eccles and Roeser, 2011). Group identification in school settings has been widely used to explain students’ sense of belonging to the school, cooperative behaviors, and psychological adaptation (Gummadam et al., 2016; Korpershoek et al., 2020). Existing research suggests that students’ identification with their school group significantly enhances their motivation, wellbeing, and mental health (Arbabi et al., 2025; Finell et al., 2024). This phenomenon is referred to as the “social cure effect,” which indicates that when individuals perceive value in their group identity and receive social support, their psychological health improves significantly (Jetten et al., 2012; Lin et al., 2024; Simonsen and Rundmo, 2020).

Social identity plays a particularly important role during adolescence. As adolescents form their self-concept and social role recognition, they often rely on their group membership to define their self-worth (Chiu and So, 2025; Paricio et al., 2020). At this stage, students are developing a sense of group identification, which not only affects their social adaptation but also has profound impacts on their psychological health and academic performance. Visual symbols within the school, such as school uniforms, emblems, and mottos, serve as tangible representations of group identity. These symbols, by visually presenting the core values and culture of the group, enhance students’ group identification.

When school uniforms are perceived as positive, inclusive, and representative symbols, they effectively stimulate students’ emotional attachment and group loyalty. Conversely, if these symbols are seen as excluding individual differences, they may lead to psychological distancing and even de-identification. Such de-identification can disrupt the emotional connection between students and their school group, hindering their psychological capital development.

At the same time, it should be noted that social identification does not increase uniformly in response to all forms of aesthetic appeal. Optimal Distinctiveness Theory (ODT) posits that individuals are motivated by two competing needs: the need for belongingness and the need for uniqueness (Brewer, 1991; Kim and Park, 2011). In institutionalized contexts such as school settings, aesthetic cues that strongly emphasize individuality may satisfy students’ self-expression needs while simultaneously weakening the salience of the uniform as a prototypical group symbol. From this perspective, certain aesthetic orientations embedded in school uniforms may activate individual-level psychological processes rather than consistently enhancing social identification.

As an important symbol of school culture and social identity, school uniforms’ aesthetic features (such as inclusivity, sustainability, and comfort) not only influence students’ aesthetic preferences but also, through their social appeal and symbolic value, activate students’ group identification and self-categorization (Craik, 2003; Turner et al., 1987). In this process, the fashion imagery of the school uniform can stimulate students’ identification with their school group, thereby promoting the formation of their psychological capital. By positively perceiving school uniforms, students reinforce their sense of belonging to the school, enhancing psychological capital dimensions such as self-efficacy, hope, resilience, and optimism. Therefore, this study hypothesizes that students, through positive perceptions of fashion imagery in school uniforms, can activate social identity, thereby further enhancing the formation of psychological capital.

In summary, Social Identity Theory provides a solid social psychological foundation for understanding the impact of fashion imagery perceptions on psychological capital. School uniforms, as external symbols, can be internalized through social identity mechanisms, thus promoting the development of students’ psychological capital. Based on Social Identity Theory, this study views school uniforms as a designable social symbol that not only affects students’ emotional connections with the school but also potentially serves as a key mechanism for fostering positive psychological resource development.

### 2.3 Psychological capital

Psychological capital (PsyCap) is a central construct in positive psychology proposed by Luthans et al. (2006). It refers to a developable positive psychological state characterized by four core dimensions: self-efficacy, hope, optimism, and resilience. Unlike relatively stable personality traits, PsyCap is conceptualized as a dynamic, malleable resource that can be cultivated, activated, and strengthened—emphasizing state-like plasticity rather than trait-like fixedness. Its theoretical foundations integrate self-efficacy theory (Bandura, 1997), hope theory (Snyder, 2000), learned optimism theory (Seligman, 2002), and the broaden-and-build theory of positive emotions (Fredrickson, 2001), jointly underscoring the developmental and socially elicitable nature of positive psychological states.

In educational settings, psychological capital is regarded as a key psychological resource that promotes students’ learning motivation, learning adaptation, and psychological resilience (Ni et al., 2025; You, 2016). Students with higher psychological capital typically exhibit greater learning engagement, stronger self-efficacy, and goal pursuit abilities, and are able to effectively regulate emotions and recover when facing academic stress. As a result, they tend to achieve better academic performance, adaptability, and mental health outcomes (Carmona-Halty et al., 2024; Fontes, 2024; Kang and Wu, 2022).

Recently, scholars have further highlighted the socially constructed attributes of PsyCap, arguing that its development depends not only on internal cognition and emotion regulation but also on external social relationships and environmental cues (Liu et al., 2025). Social Identity Theory offers a compelling framework for understanding this process. Haslam et al. (2009) proposed the notion of “social identity as a source of resilience,” suggesting that a sense of group belonging can cultivate hope, optimism, and self-efficacy. Subsequent evidence indicates that the establishment and maintenance of social identities enhance perceived social support and psychological resilience while reducing the risk of depression (Cruwys et al., 2013; Iyer et al., 2009).

Within school environments, social identification may serve as an important mediator in the development of PsyCap. As a primary site of adolescent socialization, schools shape students’ social identity through shared goals, ritualized activities, and symbolic systems (Brunsma, 2004). When students perceive the school’s image positively and identify with it, they are more likely to experience hope, optimism, and self-efficacy; conversely, a lack of identification may be associated with psychological depletion. Accordingly, social identification functions not only as a social antecedent of PsyCap but also as a psychological pathway for its development. Building on this reasoning, the present study posits that the fashion imagery of school uniforms may indirectly foster students’ psychological capital by strengthening social identification.

## 3 Methodology

**Figure 1:**
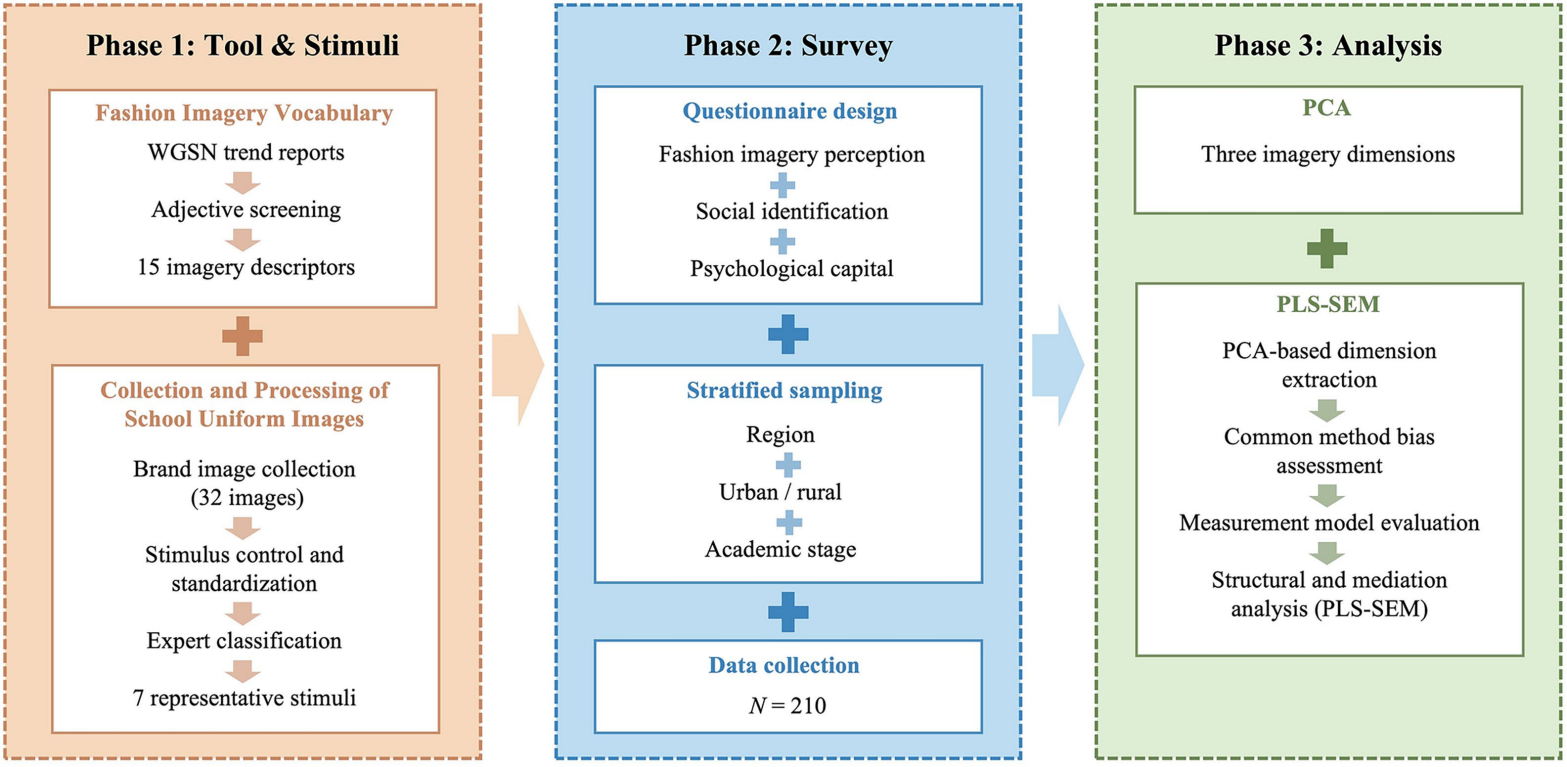
Research design and analytical procedure.

This study employed a questionnaire-based research design to examine the relationships among students’ perceptions of school-uniform fashion imagery, social identification, and psychological capital. As illustrated in Figure 1, the overall research procedure was organized into three sequential phases. Phase 1 (Tool & Stimuli): Affective adjectives were curated from mainstream fashion-trend platforms to derive core fashion-imagery dimensions, and standardized school-uniform images were prepared as visual stimuli. Phase 2 (Survey): The questionnaire measured perceptions of uniform fashion imagery, social identification, and psychological capital to probe psychological responses to style features and the underlying social-psychological mechanisms. Phase 3 (Analysis): PCA on adjective ratings reduced dimensionality and yielded the main imagery dimensions; PLS-SEM then tested relationships among imagery dimensions, social identification, and psychological capital to assess the model’s robustness and validity.

### 3.1 Measurement tool development and stimulus material preparation

#### 3.1.1 Fashion imagery vocabulary collection and screening

To accurately measure students’ perceptions of school uniform fashion imagery, this study developed a “fashion imagery measurement vocabulary”. Drawing on the work of Ge and Shaari (2023), the study extracted affective adjectives from the WGSN (Worth Global Style Network) 2026–2027 macro trend reports. These adjectives were then categorized and screened based on their semantic characteristics, ultimately resulting in 15 representative adjectives: individualistic, playful, nostalgic, creative, comfortable, safe, healthy, multifunctional, durable, practical, fashionable, inclusive, sustainable, socially responsible, and technological. These adjectives formed the initial vocabulary for measuring students’ perceptions of fashion imagery in school uniforms.

#### 3.1.2 Collection and processing of fashion school uniform images

To ensure the visual stimuli reflected current aesthetic perceptions in the Chinese school uniform market, the research team collected recent uniform images from major brands (Giuseppe, Eton Kidd, Sameite). After designer review emphasizing style diversity and representativeness, 32 images were retained.

**Figure 2:**
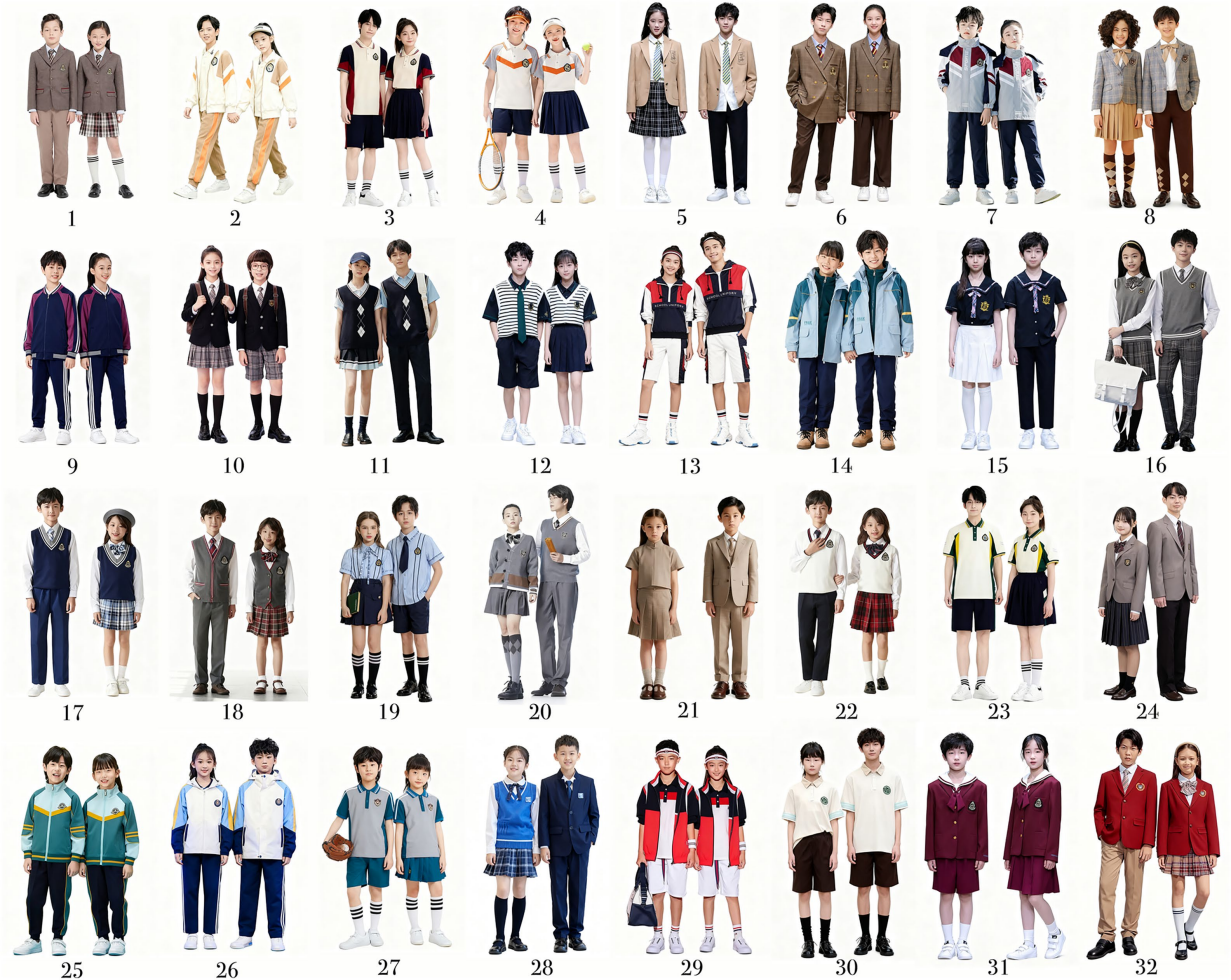
Standardized school uniform images.

During stimulus selection, all uniforms were limited to spring–autumn styles, which are widely adopted across different regions in China, and were designed to support routine school activities (e.g., classroom movement and daily walking). To control non-design factors (e.g., background, posture, expression) and maintain visual consistency, all images were standardized using AI-based editing, unifying background color, model posture, and gender (Figure 2).

**Figure 3:**
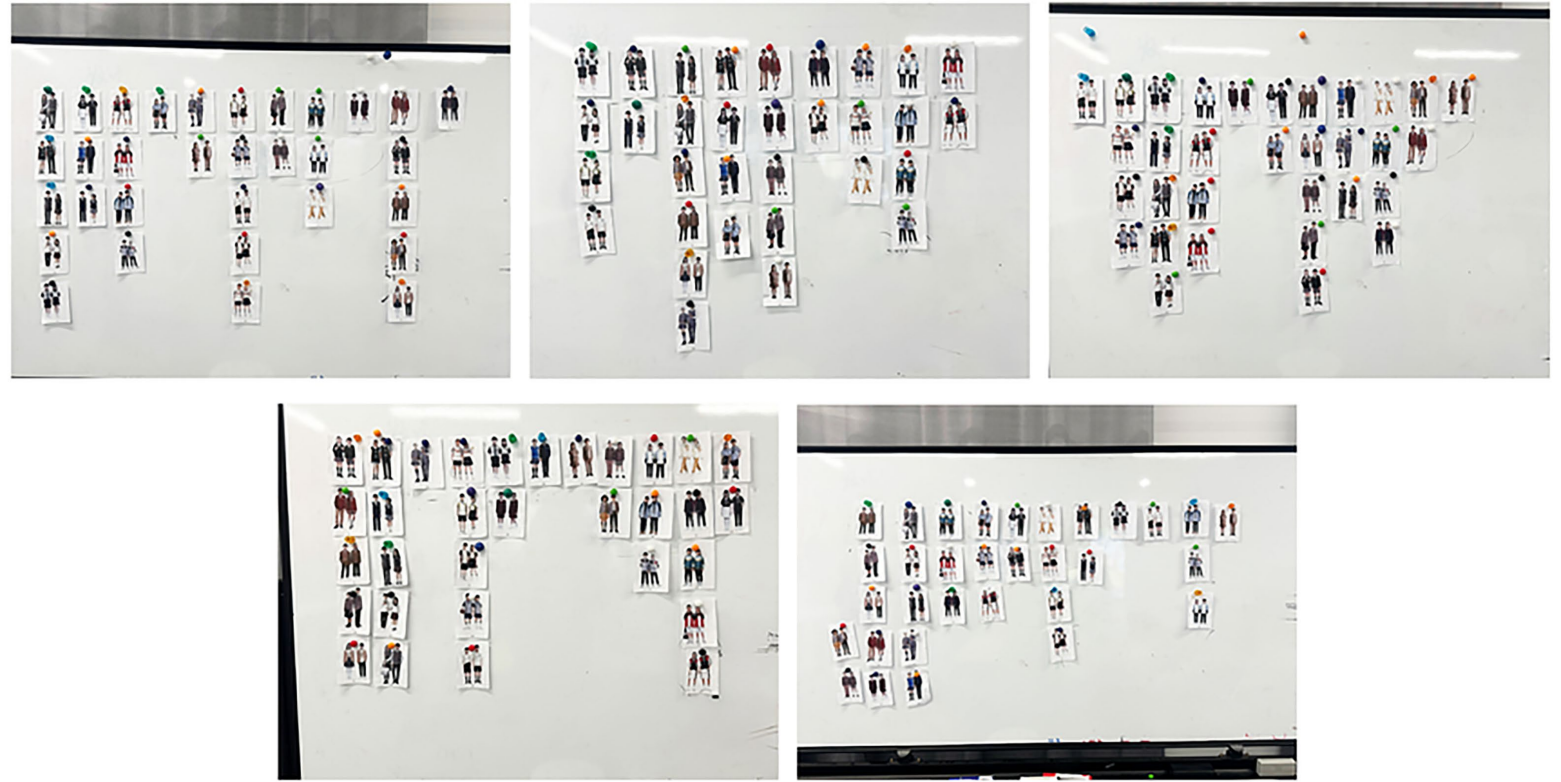
Classification results of school uniform images by experts.

**Figure 4:**
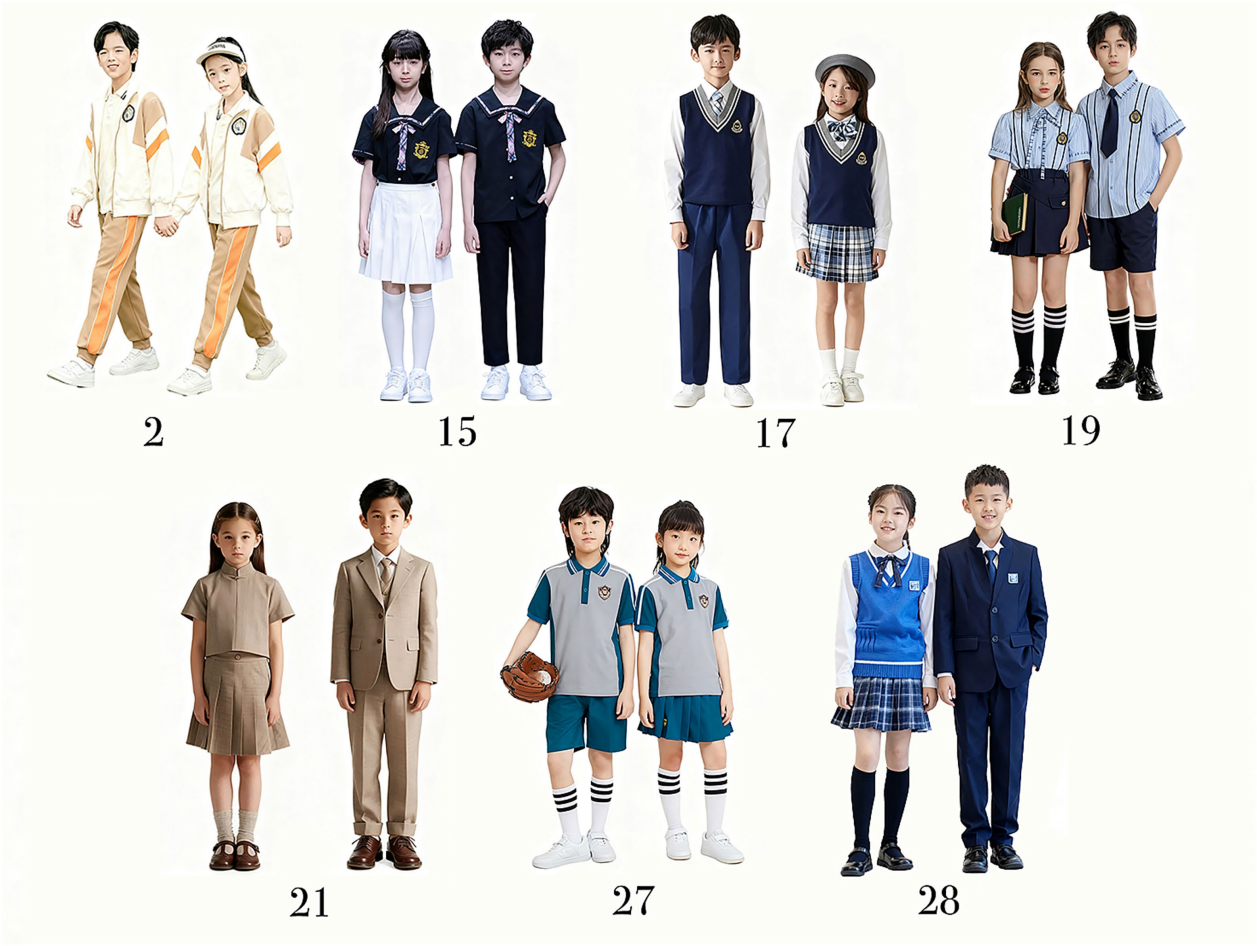
Formal experimental stimulus materials.

To ensure that the final stimuli comprehensively represented the major contemporary styles of school uniforms while avoiding redundancy and stylistic bias, an expert classification and screening process was conducted after image standardization. Five experts with extensive professional and academic experience in fashion design and aesthetics independently reviewed and categorized the 32 standardized images (Figure 3). The research team then aggregated the experts’ results and analyzed consistency to identify key style categories with high consensus. Based on these categories, the experts re-evaluated the images and selected the most representative examples of each style. Ultimately, seven images were unanimously identified as representative stimuli for the formal experiment (Figure 4).

### 3.2 Questionnaire design and data collection

#### 3.2.1 Questionnaire design

This study employed a structured questionnaire consisting of four modules:

(1) Basic Information Module: This module gathers background variables such as the participants’ gender, enrollment status, and experiences with wearing school uniforms.
(2) Fashion Imagery of School Uniforms Module: Participants are asked to subjectively assess the degree of fit between various adjectives and the style of the presented school uniforms, based on the images shown. Before responding to this section, participants were provided with a brief instruction to standardize the evaluation perspective. Specifically, participants who were currently students were asked to respond based on their current student experience, whereas participants who were not currently students were instructed to recall their own student-period experiences when evaluating the presented school uniform images. Participants were then asked to subjectively assess the degree of fit between various descriptive adjectives and the style of the presented school uniforms based on the images shown.
(3) Social Identification Module: Based on the social identity measurement tool by Hong et al. (2004), this module asks participants to select the option that most closely reflects their identity from the provided school uniform images to reflect their sense of school belonging. Participants are instructed to choose the option that best matches their personal identity based on their subjective perception of the presented school uniform images from the following four options: A. Wearing this fashionable school uniform, I feel I am a part of the school; B. Wearing this fashionable school uniform, I feel I am first a part of society, and second a part of the school; C. Wearing this fashionable school uniform, I feel I am first a part of the school, and second a part of society; D. Wearing this fashionable school uniform, I feel I am a part of society.
(4) Psychological capital, measured using the Psychological Capital Questionnaire developed by Luthans et al. (2006), encompassing self-efficacy, optimism, resilience, and hope, with additional items adapted from Li et al. (2021) and Carter (2024).

#### 3.2.2 Participants and data collection

Data were collected through an online survey administered via Credamo,^1^ a professional research platform in China. To enhance the national representativeness and external validity of the findings, a stratified sampling strategy was employed. Quotas were set based on three key dimensions: geographical region (eastern, central, western China), residential context (urban vs. rural), and educational stage (primary/secondary school vs. tertiary education), ensuring the sample captured diverse student experiences with school uniforms.

The final sample consisted of 210 valid responses (*N* = 210), with a nearly balanced gender distribution (48.0% male, 52.0% female).

All participants reported firsthand experience wearing school uniforms during their education, ensuring evaluations were grounded in actual experience rather than speculation. Although the sample included individuals who were not currently enrolled as students, the present study is mechanism-oriented, focusing on how school-uniform imagery functions as an institutionalized visual symbol that activates social identification and psychological capital. These psychological processes are shaped through repeated exposure during formative school years and reflect relatively stable, internalized identity structures, rather than momentary reactions that are primarily dependent on current enrollment status. Accordingly, retrospective evaluation anchored to participants’ student-period experiences provides a defensible basis for generalizing the observed identity-based psychological mechanisms to the target population of school students.

To validate the sufficiency of the sample size for the analytical approach—partial least squares structural equation modeling—*a priori* power analysis was conducted. Given a medium effect size (*f*^2^ = 0.15), *α* = 0.05, and a statistical power of 0.95, the minimum required sample size was 129. Additionally, following the common rule-of-thumb in PLS-SEM (10 times the number of predictors in the most complex path), the minimum requirement was 60. The obtained sample size of 210 comfortably exceeds both thresholds, ensuring adequate power for model estimation and hypothesis testing.

The study adhered strictly to established research ethics guidelines. All participants provided informed consent before participation, took part voluntarily, received modest compensation, and all responses were anonymized to protect privacy.

## 4 Data analysis

### 4.1 Principal component analysis

Exploratory factor analysis was conducted on 15 adjective variables. The KMO value (0.829) exceeded the 0.8 threshold, and Bartlett’s Test (*χ^2^* = 1552.670, df = 105, *p* < 0.001) confirmed the data’s suitability for factor analysis. Principal component analysis with varimax rotation extracted three factors (eigenvalues > 1.2) explaining 61.654% of total variance, indicating good structural validity. All loadings exceeded 0.45 without notable cross-loadings, demonstrating satisfactory convergent and discriminant validity (Table 1).

**Table 1:** Rotated factor loadings.

Constructs	Factor 1	Factor 2	Factor 3
Fashionable	0.791	−0.180	0.000
Creative	0.775	0.377	0.044
Individualistic	0.774	0.237	0.023
Energetic	0.698	0.071	0.269
Playful	0.690	0.522	0.068
Sustainable	0.090	0.820	0.182
Socially responsible	0.103	0.815	0.216
Technological	0.433	0.705	0.215
Inclusive	0.202	0.607	0.436
Nostalgic	−0.383	0.463	0.315
Practical	−0.036	0.127	0.803
Durable	−0.084	0.159	0.796
Safe	0.031	0.167	0.740
Healthy	0.251	0.243	0.609
Comfortable	0.179	0.124	0.451

Based on factor loadings and semantic interpretation, three aesthetic dimensions were identified, each reflecting a distinct mode of perceptual evaluation of school-uniform fashion imagery.

Individuality-oriented aesthetics (“individualistic,” “creative,” “fashionable,” “energetic,” “playful”) capture perceptions related to self-expression and stylistic vitality. At the perceptual level, this dimension is grounded in visual cues associated with distinctiveness and expressive variation, rather than explicit opportunities for personalization. Such cues include dynamic or contrasting color schemes, unconventional or asymmetric detailing, visually salient accents, and silhouettes that deviate from strict uniformity while remaining within institutional boundaries. Through these features, students perceive the uniform as expressive, lively, and capable of conveying individuality in appearance, forming an aesthetic judgment centered on visual dynamism and stylistic character.

Responsibility-oriented aesthetics (“inclusive,” “sustainable,” “nostalgic,” “socially responsible,” and “technological”) capture perceptions associated with collective values, social ethics, and institutional continuity. In the context of school uniforms as public and highly institutionalized forms of dress, students’ evaluations of uniform imagery are not limited to surface attractiveness or individual expression, but also incorporate a sense of participation in collective and social roles. At this level, social responsibility is transformed into a perceptible and evaluative aesthetic orientation—namely, responsibility-oriented aesthetics. Specifically, students do not interpret social responsibility through abstract ethical identification, but rather through the aesthetic interpretation of concrete visual and material cues. This responsibility-oriented aesthetic is characterized by preferences for collective values, long-term use, and rational order, and is reflected at the design level in the appreciation of sustainable materials, non-gendered or inclusive silhouettes, restrained decorative expression with functional priority, and a rational, technological aesthetic that conveys systematization and reliability. Through these stable, restrained, and orderly design expressions, students are able to translate social responsibility into an aesthetic judgment centered on appropriateness, sustainability, and institutional identification.

Function-oriented aesthetics (“healthy,” “safe,” “practical,” “durable,” “comfortable”) capture perceptions related to usability and bodily comfort. At the perceptual level, this dimension is grounded in visual cues that signal functional reliability and physical ease rather than symbolic meaning. These cues include relaxed or ergonomic silhouettes, visually breathable or well-structured constructions, soft or flexible material appearances, and design features suggesting protection, stability, and freedom of movement. Based on such cues, students infer comfort, safety, and practicality directly from the uniform’s appearance, forming an aesthetic evaluation rooted in anticipated wearability and everyday bodily experience.

Together, these three dimensions capture students’ perceptions of school-uniform fashion imagery across individual-expressive, collective-symbolic, and functional-experiential levels.

### 4.2 Common method bias

Given that all variables were collected using a self-report questionnaire at a single point in time, the potential influence of common method bias (CMB) was carefully examined. Firstly, several procedural remedies were implemented during the data collection stage to mitigate common method bias (Podsakoff et al., 2003). Specifically, participation was anonymous, respondents were informed that there were no right or wrong answers, and the questionnaire was administered for academic research purposes only. In addition, AI-based image standardization was employed to minimize the influence of irrelevant visual cues, thereby reducing systematic measurement noise. Secondly, Harman’s single-factor test was conducted by performing an unrotated exploratory factor analysis on all measurement items. The results showed that five factors with eigenvalues greater than 1 were extracted, and the first principal component accounted for 38.026% of the total variance, which is below the commonly accepted threshold of 40%, indicating that no significant common method bias was present in this study. Thirdly, following recommendations for PLS-SEM analysis, a full collinearity assessment was conducted by examining variance inflation factors (VIFs) for all latent constructs (Kock, 2015). All VIF values were below the conservative threshold of 3.3, suggesting that common method bias was unlikely to be a serious concern in this study.

### 4.3 Measurement model

This study employed SmartPLS 4.1 software to conduct Partial Least Squares Structural Equation Modeling (PLS-SEM). To assess the internal consistency and convergent validity of the measurement model, Cronbach’s *α*, composite reliability (CR), factor loadings, and Average Variance Extracted (AVE) were examined for all multi-item constructs. The results are presented in Table 2. All multi-item constructs demonstrated satisfactory reliability and convergent validity, with Cronbach’s *α* and CR values exceeding the recommended threshold of 0.70 and AVE values above 0.50 (Hair et al., 2017).

**Table 2:** Measurement model evaluation results.

Constructs	Items	Outer loadings	Cronbach’s *α*	Composite reliability	AVE
Psychological capital (PC)	Hope 1	0.795	0.948	0.955	0.639
	Hope 2	0.790			
	Hope 3	0.840			
	Optimism 1	0.872			
	Optimism 2	0.830			
	Optimism 3	0.870			
	Resilience 1	0.890			
	Resilience 2	0.859			
	Resilience 3	0.899			
	Self-Efficacy 1	0.915			
	Self-Efficacy 2	0.833			
	Self-Efficacy 3	0.841			
Responsibility-oriented aesthetics (RA)	Inclusive	0.720	0.806	0.865	0.567
	Sustainable	0.815			
	Nostalgic	0.558			
	Socially responsible	0.799			
	Technological	0.837			
Individuality-oriented aesthetics (IA)	Individualistic	0.827	0.848	0.892	0.625
	Creative	0.885			
	Fashionable	0.713			
	Energetic	0.706			
	Playful	0.808			
Function-oriented aesthetics (FA)	Healthy	0.744	0.758	0.835	0.504
	Safe	0.706			
	Practical	0.717			
	Durable	0.633			
	Comfortable	0.745			

For constructs that are conceptually unidimensional and perceptual in nature, a parsimonious measurement approach was adopted in this study. Specifically, social identification was intentionally operationalized as a single-item construct designed to capture participants’ overall sense of affiliation with the school. This construct reflects a global and intuitive identity judgment rather than a multidimensional attitudinal structure. Consistent with prior methodological research, single-item measurement is considered appropriate when a construct is concrete, clearly defined, and readily understood by respondents (Bergkvist and Rossiter, 2007).

From a methodological perspective, variance-based structural equation modeling approaches, such as partial least squares structural equation modeling (PLS-SEM), permit the inclusion of single-item constructs and do not require all variables in the model to be specified as latent constructs with multi-item measurement models (Hair et al., 2019). Accordingly, in the present study, social identification was treated as an observed variable and used exclusively for estimating structural path relationships in the mediation analysis. Reliability and validity indices such as Cronbach’s alpha, composite reliability, and average variance extracted were therefore not computed for this construct, as these indices are not applicable to single-item measures.

Discriminant validity among the multi-item constructs was assessed using the Fornell–Larcker criterion (Fornell and Larcker, 1981) and the Heterotrait–Monotrait Ratio (HTMT). The square root of AVE for each construct exceeded its correlations with other constructs, and all HTMT values were below the conservative threshold of 0.85, indicating satisfactory discriminant validity (Table 3).

**Table 3:** Discriminant validity.

Fornell-Larcker criterion	IA	FA	PC	GI	RI
IA	0.791				
FA	0.249	0.710			
PC	0.476	0.557	0.800		
GI	−0.220	−0.164	−0.047	1.000	
RI	0.431	0.539	0.463	−0.395	0.753

HTMT criterion	IA	FA	PC	GI	RI
IA					
FA	0.329				
PC	0.531	0.630			
GI	0.252	0.194	0.102		
RI	0.563	0.708	0.518	0.412	

### 4.4 Model development

This study adopts Social Identity Theory (SIT) and Psychological Capital Theory (PsyCap Theory) as the framework to explore the mechanisms through which different types of school uniform fashion imagery affect students’ psychological capital. The hypothesized model is grounded in the assumption that students’ perceptions of school uniform imagery operate at multiple psychological levels, which is consistent with the three aesthetic dimensions emerging from the principal component analysis. The logical flow is as follows:

Firstly, responsibility-oriented aesthetics reflect value-oriented design features such as inclusiveness, sustainability, and social responsibility. This dimension captures students’ tendency to interpret certain visual features of school uniforms as symbolic representations of institutional norms and collective values. These collective-oriented symbols evoke students’ sense of shared values and group belonging. Prior research has demonstrated that when visual designs or symbolic cues convey collective values and group identity, individuals’ sense of identification increases significantly, providing a social–psychological foundation for the accumulation of positive psychological resources (Guegan et al., 2017). Accordingly, when students perceive school uniforms as embodying social responsibility and cultural continuity, their social identification strengthens, thereby enhancing psychological capital.

Secondly, individuality-oriented aesthetics capture students’ pursuit of self-expression, creativity, and distinctiveness. This aesthetic orientation reflects a perceptual focus on how school uniforms allow—or constrain—the expression of personal identity within a standardized context. On the one hand, individuals express self-worth through personalized clothing symbols, thereby reinforcing self-efficacy and positive psychological states (Roster, 2024). On the other hand, because school uniforms function as prototypical group symbols, an emphasis on individuality may limit their effectiveness in activating social identification. Therefore, individuality-oriented aesthetics are expected to influence psychological capital primarily through a direct, self-focused pathway rather than via social identification.

Finally, function-oriented aesthetics emphasizes comfort, safety, and practicality in clothing. Unlike the other two dimensions, this orientation is less symbolic and more closely related to students’ embodied experience and daily usability. From an experiential perspective, perceptions of the physical comfort and safety of school uniforms directly influence students’ psychological experiences, enhancing their sense of environmental fit and control over the school environment (Reidy, 2021). These experiential states are closely linked to self-efficacy, emotional security, and resilience—core components of psychological capital (Bandura, 1997). Importantly, because such effects are grounded in direct bodily and affective experience rather than symbolic meaning, they do not necessarily depend on processes of social identification. Accordingly, this study posits that function-oriented aesthetics are directly associated with psychological capital, independent of social identification.

Taken together, the three school-uniform aesthetic orientations can be understood as relatively independent and stable dimensions not because of arbitrary categorization by the researcher, but because they are rooted in students’ multi-level processes of meaning construction within an institutional educational context. As a highly institutionalized form of everyday dress, school uniforms simultaneously embody normative values, regulate identity expression, and shape embodied experiences. Consequently, their aesthetic meanings naturally differentiate into distinct yet interrelated evaluative pathways at the cognitive and experiential levels. From this perspective, responsibility-oriented, individuality-oriented, and function-oriented aesthetics are not merely different facets of a single aesthetic judgment; rather, they represent concrete manifestations of the multidimensional meaning structure of school uniforms as perceived by students.

**Figure 5:**
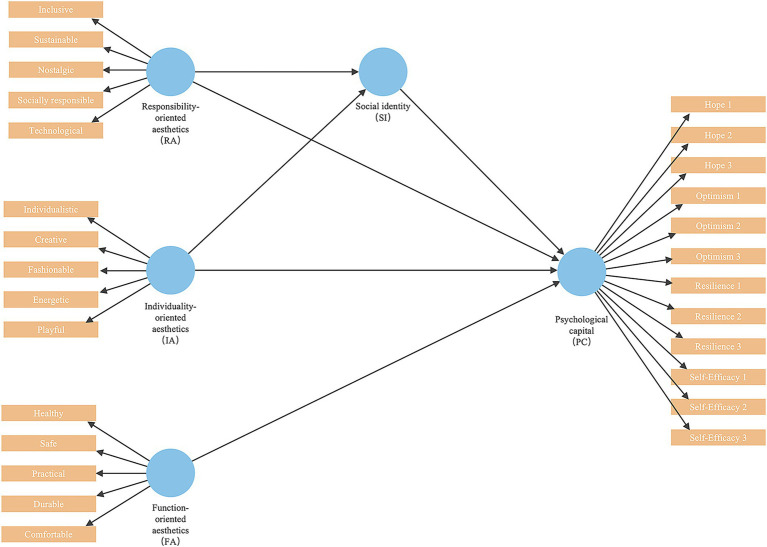
Conceptual model.

Building on this conceptualization, the present study further proposes that these three aesthetic orientations contribute to psychological capital through differentiated psychological pathways. Specifically, responsibility-oriented aesthetics primarily operate by activating shared values and collective identification, individuality-oriented aesthetics exert their influence through self-expression and self-related identification processes, and function-oriented aesthetics affect psychological capital via comfort, safety, and perceived practicality. Together, these distinct yet complementary pathways constitute the theoretical basis of the hypothesized model (Figure 5).

### 4.5 Structural model

**Figure 6:**
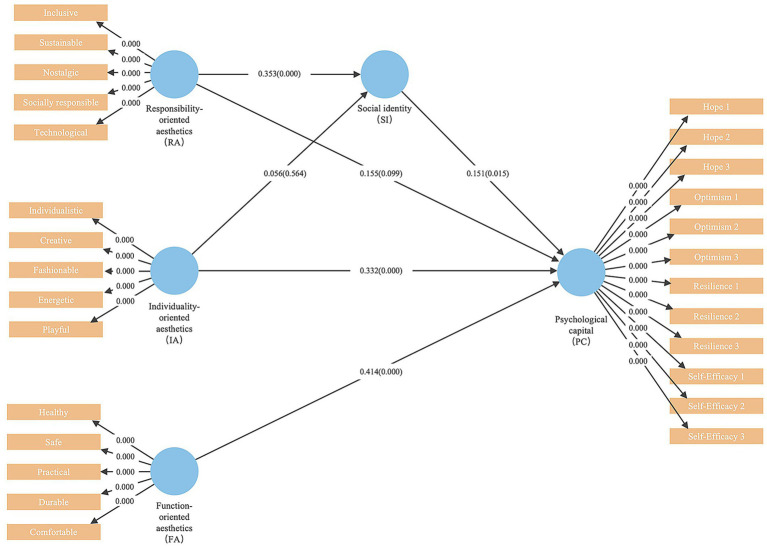
Structural modeling result.

The study used the bootstrapping resampling method with 5,000 repetitions to conduct statistical inference on the significance of the model coefficients. The corresponding results are shown in Figure 6.

Direct path analysis results indicated that Individuality-oriented Aesthetics (*b* = 0.332, *t* = 3.734, *p* < 0.001, 95% CI [0.157, 0.507]), Function-oriented Aesthetics (*b* = 0.414, *t* = 4.988, *p* < 0.001, 95% CI [0.251, 0.577]), and Social identification (*b* = 0.151, *t* = 2.443, *p* = 0.015, 95% CI [0.029, 0.273]) all had significant positive effects on Psychological Capital. However, the direct positive effect of Responsibility-oriented Aesthetics on Psychological Capital was marginally significant (*b* = 0.155, *t* = 1.650, *p* = 0.099, 95% CI [−0.029, 0.339]). Additionally, Responsibility-oriented Aesthetics had a significant positive effect on Social identification (*b* = 0.353, *t* = 4.989, *p* < 0.001, 95% CI [0.214, 0.492]), while the effect of Individuality-oriented Aesthetics on Social identification was not significant (*b* = 0.056, *t* = 0.577, *p* = 0.564, 95% CI [−0.246, 0.134]).

Mediation analysis revealed that Social identification played a significant mediating role between Responsibility-oriented Aesthetics and Psychological Capital (*b* = 0.053, *t* = 2.074, *p* = 0.038, 95% CI [0.002, 0.104]). As the direct effect of Responsibility-oriented Aesthetics on Psychological Capital was positive (marginally significant), and the indirect effect through Social identification was also positive with a confidence interval that did not include zero, it indicates that Social identification plays a significant mediating role. However, the mediating effect of Social identification between Individuality-oriented Aesthetics and Psychological Capital was not significant (*b* = 0.008, *t* = 0.567, *p* = 0.571, 95% CI [−0.021, 0.037]), and this path was not supported by the data.

The model’s explanatory power for the endogenous variables was evaluated using *R*^2^. The results showed that the model explained 26.0% of the variance in Social identification (*R*^2^ = 0.260) and 41.5% of the variance in Psychological Capital (*R*^2^ = 0.415). According to the criteria set by Chin (1998), this indicates that the theoretical model developed in this study has a moderate to strong predictive power for Social identification and Psychological Capital.

## 5 Discussion

This study examined how students’ perceptions of fashion imagery in school uniforms influence the development of psychological capital through social identity. The findings indicate that responsibility-oriented aesthetics strengthen students’ social identity, which subsequently contributes to the accumulation of psychological capital. When school uniforms display visual characteristics such as inclusivity, sustainability, and social responsibility, students are more likely to perceive a shared system of values through their aesthetic experience, thereby reinforcing their sense of belonging and emotional connection to the school. This result aligns with the core proposition of Social Identity Theory: individuals derive meaning and value affirmation from group membership, which enhances wellbeing, hope, and resilience (Cruwys et al., 2013; Iyer et al., 2009). Thus, responsibility-oriented aesthetics function not only as visual design elements but also as socio-psychological signals that are associated with positive psychological development among students.

In contrast, individuality-oriented aesthetics do not significantly influence social identity. Rather than indicating a weak or null effect, this non-significant result reflects a distinct psychological mechanism. Such aesthetic orientations primarily communicate personal traits, creativity, and distinctiveness, which are more closely related to individual self-expression than to the reinforcement of collective group boundaries. When exposed to personalized school uniform designs, students are therefore more likely to gain individual psychological benefits—such as enhanced self-efficacy or a sense of creativity—rather than increased group-level identification. This pattern can be understood through Optimal Distinctiveness Theory, which suggests that individuals seek a balance between belonging and uniqueness. In the context of school uniforms, an emphasis on individuality may support self-definition but weaken the uniform’s function as a prototypical group symbol, thereby limiting its capacity to activate social identification. Consistent with this interpretation, individuality-oriented aesthetics show a significant direct effect on psychological capital but no indirect effect via social identity, indicating a primarily self-focused rather than group-mediated pathway.

Moreover, functional aesthetics exert a significant positive effect on psychological capital. Although this effect does not operate through social identity, it is mainly driven by the psychological safety and positive emotions arising from comfort and security. Thoughtful design of school uniforms in terms of materials and structure can enhance students’ sense of environmental fit, boosting their confidence, optimism, and resilience (Kühner et al., 2024; Luthans et al., 2006). This finding suggests that school uniforms are not only cultural symbols but also psychological support media; their functional attributes help create positive physical and emotional experiences that underpin the formation of psychological capital.

Taken together, the three aesthetic dimensions reflect complementary psychological pathways through which school uniform design contributes to students’ psychological capital. Responsibility-oriented aesthetics function at a collective-symbolic level by strengthening shared values and social identification; individuality-oriented aesthetics operate at a personal level by supporting self-expression and individual psychological resources; and function-oriented aesthetics act at a contextual level by fostering comfort, safety, and environmental control. This integrated perspective suggests that psychological capital in educational settings is not activated through a single mechanism, but is jointly shaped by group affiliation, self-definition, and embodied experience. By moving beyond isolated aesthetic effects, this study provides a more nuanced understanding of how design features of school uniforms can simultaneously support students’ social integration and psychological development.

### 5.1 Theoretical implications

This study contributes to theory by integrating design psychology and positive psychology to explain how different aesthetic orientations embedded in school uniforms relate to psychological capital through distinct psychological pathways. Rather than assuming a uniform role of aesthetics, the findings highlight the conditional and differentiated nature of identity-based mechanisms in institutional design contexts.

Firstly, this study extends Social Identity Theory by embedding it within the aesthetic context of school uniforms. While prior research has primarily conceptualized social identity as emerging from interpersonal interaction or cultural affiliation (e.g., Bentley et al., 2020; Verkuyten, 2021), the present findings demonstrate that value-oriented aesthetic cues—such as inclusivity, sustainability, and social responsibility—can activate shared-value orientations and strengthen social identification. At the same time, the non-significant effect of individuality-oriented aesthetics on social identification reveals an important boundary condition of Social Identity Theory in institutional dress settings. Specifically, drawing on Optimal Distinctiveness Theory (Brewer, 1991), this study suggests that school uniforms constitute a highly institutionalized and norm-regulated context in which collective conformity is structurally emphasized. In such contexts, aesthetic cues that strongly signal individuality and personal uniqueness may satisfy self-expression needs but simultaneously reduce the salience of group prototypicality. As a result, individuality-oriented aesthetics tend to activate self-definition processes rather than group-based identification. This finding highlights a theoretical tension between individuality and collective conformity and explains why personalization in school uniform aesthetics does not automatically translate into stronger social identification.

Secondly, this study advances psychological capital theory in educational research by highlighting the role of situational and environmental triggers. Whereas prior studies in positive psychology have largely focused on individual traits and psychological interventions (Kang and Wu, 2022; Ni et al., 2025), the present research demonstrates that aesthetic characteristics of clothing can shape psychological capital through multiple pathways. Importantly, these pathways differ in their reliance on social identity: responsibility-oriented aesthetics operate primarily through identity-based mechanisms, whereas individuality-oriented and function-oriented aesthetics influence psychological capital through self-focused and experience-based mechanisms, respectively.

Finally, by proposing and empirically testing a model linking “aesthetic perception—social identity—psychological capital,” this study offers a more nuanced theoretical account of how design influences psychological states. The differentiated pathways identified in this model underscore that aesthetic personalization and collective identity are not inherently aligned, particularly in institutional contexts. This insight enriches theoretical discussions on social identity, optimal distinctiveness, and design psychology, and clarifies when and why identity-based mediation mechanisms may succeed or fail.

### 5.2 Practical implications

This study provides several practical implications for educational administrators, teachers, and school uniform designers. Educational policymakers can incorporate the findings of this study into school uniform reform and campus culture development by promoting design principles that emphasize inclusivity, sustainability, and social responsibility, thus fostering students’ psychological wellbeing and socio-emotional development, particularly enhancing their psychological capital, such as self-efficacy, hope, resilience, and optimism.

At the school level, uniforms can be used not only as external clothing but also as a medium for aesthetic and psychological intervention. Activities such as student-designer co-creation or school-wide uniform showcases can strengthen students’ sense of belonging and aesthetic engagement, thus activating positive psychological resources and promoting students’ psychological growth and the accumulation of psychological capital.

Teachers and designers can integrate positive psychology and Social Identity Theory into aesthetic education and design practices, drawing on the experiences from Psychological Capital Interventions (PCI) to cultivate students’ hope, confidence, and resilience. Furthermore, school uniforms should be seen not merely as institutional symbols but as important psychological and aesthetic media that enhance students’ group identity, psychological wellbeing, and psychological capital, thereby supporting students’ psychological development and the formation of their psychological capital.

### 5.3 Limitations and future research

Despite its innovative contributions, this study has several limitations. Firstly, as it employed a cross-sectional survey design, causal relationships among variables could not be fully established. Future research could adopt longitudinal or experimental approaches—such as randomized assignments of uniform styles or group identity priming—to strengthen causal inference. In addition, although all participants had firsthand experience of wearing school uniforms, future studies could further extend and validate the present findings by employing samples of currently enrolled students or by adopting longitudinal designs to track changes in social identification and psychological capital over time.

Secondly, as the sample was drawn primarily from a Chinese cultural context, variations in the interpretation of descriptors such as “inclusive,” “individualistic,” and “playful” may exist across regions. Future studies should test the generalizability and cultural adaptability of the model using cross-cultural samples and further examine the moderating role of cultural context.

## Data Availability

The original contributions presented in the study are included in the article/supplementary material, further inquiries can be directed to the corresponding author.

## Footnotes

1
https://www.credamo.com

## References

[ref1] Abrams D. (2009). Social identity on a national scale: optimal distinctiveness and young people’s self-expression through musical preference. Group Process. Intergroup Relat. 12, 303–317. doi: 10.1177/1368430209102841

[ref2] Alton J. Cimpian A. Butler L. P. (2025). The role of gender labels and gendered appearances in children’s social inferences. Cognition 265:106281. doi: 10.1016/j.cognition.2025.106281, 40829329

[ref3] Ansari A. Shepard M. Gottfried M. A. (2022). School uniforms and student behavior: is there a link? Early Child. Res. Q. 58, 278–286. doi: 10.1016/j.ecresq.2021.09.012, 35068671 PMC8775910

[ref4] Arbabi K. Yeh C. J. Sangkar P. R. (2025). Students’ perceptions of factors contributing to a happy school environment in Iran. Child Youth Care Forum 54, 735–754. doi: 10.1007/s10566-024-09839-z

[ref5] Badaoui K. Lebrun A.-M. Su C.-J. Bouchet P. (2018). The influence of personal and social identity on the clothing consumption of adolescents. Can. J. Admin. Sci./Revue Canadienne des Sciences de l'Administration 35, 65–78. doi: 10.1002/cjas.1397

[ref6] Bandura A. (1997). Self-efficacy: the exercise of control. New York, NY: W H Freeman/Times Books/Henry Holt & Co., ix, 604.

[ref7] Beaudoin P. Lachance M. J. (2006). Determinants of adolescents’ brand sensitivity to clothing. Fam. Consum. Sci. Res. J. 34, 312–331. doi: 10.1177/1077727X06286418

[ref8] Bentley S. V. Greenaway K. H. Haslam S. A. Cruwys T. Steffens N. K. Haslam C. . (2020). Social identity mapping online. J. Pers. Soc. Psychol. 118:213. doi: 10.1037/pspa0000174, 31556682

[ref9] Bergkvist L. Rossiter J. R. (2007). The predictive validity of multiple-item versus single-item measures of the same constructs. J. Mark. Res. 44, 175–184. doi: 10.1509/jmkr.44.2.175

[ref10] Brewer M. B. (1991). The social self: on being the same and different at the same time. Personal. Soc. Psychol. Bull. 17, 475–482. doi: 10.1177/0146167291175001

[ref11] Briffett-Aktaş C. Ying J. (2025). Expressions of educational change agents: student voice influences in higher education. High. Educ., 1–13. doi: 10.1007/s10734-025-01472-6

[ref12] Brunsma D. L. (2004). The school uniform movement and what it tells us about American education: a symbolic crusade. Lanham, MD: R&L Education.

[ref13] Camus L. Rajendran G. Stewart M. E. (2024). Social self-efficacy and mental well-being in autistic adults: exploring the role of social identity. Autism 28, 1258–1267. doi: 10.1177/13623613231195799, 37728250 PMC11067414

[ref14] Carmona-Halty M. Alarcón-Castillo K. Semir-González C. Sepúlveda-Páez G. Mena-Chamorro P. Barrueto-Opazo F. . (2024). How study-related positive emotions and academic psychological capital mediate between teacher-student relationship and academic performance: a four-wave study among high school students. Front. Psychol. 15:1419045. doi: 10.3389/fpsyg.2024.1419045, 39268383 PMC11390622

[ref15] Carter J. W. (2024). Enhancing student performance in business simulation games through psychological capital and flow. Int. J. Manag. Educ. 22:101031. doi: 10.1016/j.ijme.2024.101031

[ref8001] Chin J. W. (1998). The partial least squares approach to structural equation modeling. Modern methods for business research. ed. Marcoulides G. A. Mahwah, NJ: Lawrence Erlbaum Associates. 295–336.

[ref16] Chiu S. W. K. So W. W. M. (2025). STEM career aspiration: does students’ social identity matter? Asia Pac. J. Educ. 45, 278–295. doi: 10.1080/02188791.2022.2108758

[ref17] Craik J. (2003). The face of fashion: cultural studies in fashion. London: Routledge.

[ref18] Cruwys T. Dingle G. A. Haslam C. Haslam S. A. Jetten J. Morton T. A. (2013). Social group memberships protect against future depression, alleviate depression symptoms and prevent depression relapse. Soc. Sci. Med. 98, 179–186. doi: 10.1016/j.socscimed.2013.09.013, 24331897

[ref19] Dagalp I. Hartmann B. J. (2022). From “aesthetic” to aestheticization: a multi-layered cultural approach. Consum. Mark. Cult. 25, 1–20. doi: 10.1080/10253866.2021.1935900

[ref20] Dewey J. (1986). Experience and education. Educ. Forum 50, 241–252. doi: 10.1080/00131728609335764

[ref21] Eccles J. S. Roeser R. W. (2011). Schools as developmental contexts during adolescence. J. Res. Adolesc. 21, 225–241. doi: 10.1111/j.1532-7795.2010.00725.x

[ref22] Edalat A. Hu R. Patel Z. Polydorou N. Ryan F. Nicholls D. (2025). Self-initiated humour protocol: a pilot study with an AI agent. Front. Digit. Health 7:1530131. doi: 10.3389/fdgth.2025.1530131, 40182587 PMC11965911

[ref23] Finell E. Tolvanen A. Shuttleworth I. Durrheim K. Vuorenmaa M. (2024). The identification environment matters: students’ social identification, perceived physical school environment, and anxiety – a cross-level interaction model. Br. J. Soc. Psychol. 63, 429–452. doi: 10.1111/bjso.12686 37747119

[ref24] Fontes A. (2024). Psychological capital promotion in universities. Eur. J. Pub. Health 34:ckae144.2267. doi: 10.1093/eurpub/ckae144.2267

[ref25] Fornell C. Larcker D. F. (1981). Structural equation models with unobservable variables and measurement error: algebra and statistics. J. Mark. Res. 18, 382–388. doi: 10.2307/3150980

[ref26] Fredrickson B. L. (2001). The role of positive emotions in positive psychology. The broaden-and-build theory of positive emotions. Am. Psychol. 56, 218–226. doi: 10.1037/0003-066x.56.3.218, 11315248 PMC3122271

[ref27] Friedrich J. Shanks R. (2023). ‘The prison of the body’: school uniforms between discipline and governmentality. Discourse Stud. Cult. Polit. Educ. 44, 16–29. doi: 10.1080/01596306.2021.1931813

[ref28] Ge B. Shaari N. (2023). Optimize the online shopping title of men’s plain-color shirts in e-commerce based on Kansei engineering. J. Glob. Fash. Mark. 14, 226–242. doi: 10.1080/20932685.2022.2085598

[ref29] Greenaway K. H. Haslam S. A. Cruwys T. Branscombe N. R. Ysseldyk R. Heldreth C. (2015). From “we” to “me”: group identification enhances perceived personal control with consequences for health and well-being. J. Pers. Soc. Psychol. 109, 53–74. doi: 10.1037/pspi0000019 25938701

[ref30] Guegan J. Segonds F. Barré J. Maranzana N. Mantelet F. Buisine S. (2017). Social identity cues to improve creativity and identification in face-to-face and virtual groups. Comput. Hum. Behav. 77, 140–147. doi: 10.1016/j.chb.2017.08.043

[ref31] Gummadam P. Pittman L. D. Ioffe M. (2016). School belonging, ethnic identity, and psychological adjustment among ethnic minority college students. J. Exp. Educ. 84, 289–306. doi: 10.1080/00220973.2015.1048844

[ref32] Hair J. F. Hult G. T. M. Ringle C. M. Sarstedt M. (2017). A primer on partial least squares structural equation modeling (PLS-SEM). Second Edn. Los Angeles: SAGE.

[ref33] Hair J. F. Risher J. J. Sarstedt M. Ringle C. M. (2019). When to use and how to report the results of PLS-SEM. Eur. Bus. Rev. 31, 2–24. doi: 10.1108/EBR-11-2018-0203

[ref34] Hammill J. Nguyen T. Henderson F. (2022). Student engagement: the impact of positive psychology interventions on students. Act. Learn. High. Educ. 23, 129–142. doi: 10.1177/1469787420950589

[ref35] Haslam S. Jetten J. Postmes T. Haslam C. (2009). Social identity, health and well-being: an emerging agenda for applied psychology. Appl. Psychol. 58, 1–23. doi: 10.1111/j.1464-0597.2008.00379.x

[ref36] He W. Luo H. Zhang D. Zhang Y. (2024). Student’s subjective feelings during classroom learning. Learn. Instr. 91:101891. doi: 10.1016/j.learninstruc.2024.101891

[ref37] Hester N. Hehman E. (2023). Dress is a fundamental component of person perception. Personal. Soc. Psychol. Rev. 27, 414–433. doi: 10.1177/10888683231157961, 36951208 PMC10559650

[ref38] Hong Y. Y. Coleman J. Chan G. Wong R. Chiu C. Y. Hansen I. . (2004). Predicting intergroup bias: the interactive effects of implicit theory and social identity. Pers. Soc. Psychol. Bull. 30, 1035–1047. doi: 10.1177/0146167204264791, 15257787

[ref39] Iyer A. Jetten J. Tsivrikos D. Postmes T. Haslam S. A. (2009). The more (and the more compatible) the merrier: multiple group memberships and identity compatibility as predictors of adjustment after life transitions. Br. J. Soc. Psychol. 48, 707–733. doi: 10.1348/014466608X397628, 19200408

[ref40] Jenkinson J. (2020). “Wear your identity”: styling identities of youth through dress–a conceptual model. Fashion Style Popul. Cult. 7, 73–99. doi: 10.1386/fspc_00006_1

[ref41] Jetten J. Haslam C. Alexander S. H. (2012). The social cure: identity, health and well-being. Hove: Psychology Press.

[ref42] Kang X. Wu Y. (2022). Investigating the linkage between school psychological capital and achievement emotions in secondary school mathematics. Asia Pac. Educ. Res. 31, 739–748. doi: 10.1007/s40299-021-00623-4

[ref43] Kim J. Park H. S. (2011). The effect of uniform virtual appearance on conformity intention: social identity model of deindividuation effects and optimal distinctiveness theory. Comput. Human Behav. 27, 1223–1230. doi: 10.1016/j.chb.2011.01.002

[ref44] Knox S. (2022). Fostering student engagement in virtual entrepreneurship education environments. Int. J. Manag. Educ. 20:100705. doi: 10.1016/j.ijme.2022.100705

[ref45] Kock N. (2015). Common method bias in PLS-SEM: a full collinearity assessment approach. Int. J. E-Collabor. 11, 1–10. doi: 10.4018/ijec.2015100101

[ref46] Korpershoek H. Canrinus E. T. Fokkens-Bruinsma M. de Boer H. (2020). The relationships between school belonging and students’ motivational, social-emotional, behavioural, and academic outcomes in secondary education: a meta-analytic review. Res. Pap. Educ. 35, 641–680. doi: 10.1080/02671522.2019.1615116

[ref47] Kühner C. Stein M. Zacher H. (2024). A person-environment fit approach to environmental sustainability in the workplace. J. Environ. Psychol. 95:102270. doi: 10.1016/j.jenvp.2024.102270

[ref48] Li Z. Yu Z. Huang S. Sam Zhou J. Yu M. . (2021). The effects of psychological capital, social capital, and human capital on hotel employees’ occupational stress and turnover intention. Int. J. Hosp. Manag. 98:103046. doi: 10.1016/j.ijhm.2021.103046

[ref49] Lin S. Mastrokoukou S. Longobardi C. Bozzato P. (2024). The influence of resilience and future orientation on academic achievement during the transition to high school: the mediating role of social support. Int. J. Adolesc. Youth 29:2312863. doi: 10.1080/02673843.2024.2312863

[ref50] Liu X.-Y. Yu C. Zhu E. Yin M. (2025). How teachers’ emotional display and emotional labor influence the relationship between students’ intrinsic learning motivation and mind wandering in class. Asia Pac. Educ. Res. 34, 739–751. doi: 10.1007/s40299-024-00892-9

[ref51] Luthans F. Youssef C. M. Avolio B. J. (2006). Psychological capital: developing the human competitive edge. New York: Oxford University Press.

[ref52] Makaremi N. Yildirim S. Morgan G. T. Touchie M. F. Jakubiec J. A. Robinson J. B. (2024). Impact of classroom environment on student wellbeing in higher education: review and future directions. Build. Environ. 265:111958. doi: 10.1016/j.buildenv.2024.111958

[ref53] Nagamachi M. (1995). Kansei engineering: a new ergonomic consumer-oriented technology for product development. Int. J. Ind. Ergon. 15, 3–11. doi: 10.1016/0169-8141(94)00052-5 12164511

[ref54] Ni J. Shi Y. Sun J. Wu Q. (2025). The effect of self-esteem on college students’ learning adaptation: a chain mediation analysis of self-efficacy and learning burnout. Asia Pac. Educ. Res. 34, 1321–1330. doi: 10.1007/s40299-024-00945-z

[ref55] Norman D. (2007). Emotional design: why we love (or hate) everyday things. New York: Basic Books.

[ref56] Paricio D. Herrera M. Rodrigo M. F. Viguer P. (2020). Association between group identification at school and positive youth development: moderating role of rural and urban contexts. Front. Psychol. 11:1971. doi: 10.3389/fpsyg.2020.01971, 32849154 PMC7427468

[ref57] Piacentini M. Mailer G. (2004). Symbolic consumption in teenagers’ clothing choices. J. Consum. Behav. 3, 251–262. doi: 10.1002/cb.138

[ref58] Podsakoff P. M. MacKenzie S. B. Lee J.-Y. Podsakoff N. P. (2003). Common method biases in behavioral research: a critical review of the literature and recommended remedies. J. Appl. Psychol. 88:879. doi: 10.1037/0021-9010.88.5.879, 14516251

[ref59] Reidy J. (2021). Reviewing school uniform through a public health lens: evidence about the impacts of school uniform on education and health. Public Health Rev. 42:1604212. doi: 10.3389/phrs.2021.1604212, 34692181 PMC8386814

[ref60] Roster C. A. (2024). Effects of personal values and clothing style confidence on consumers’ interest in upcycled clothing products. Sustainability 16:6393. doi: 10.3390/su16156393

[ref61] Sabic-El-Rayess A. Mansur N. N. Batkhuyag B. Otgonlkhagva S. (2020). School uniform policy’s adverse impact on equity and access to schooling. Compare 50, 1122–1139. doi: 10.1080/03057925.2019.1579637

[ref62] Seligman M. E. P. (2002). Authentic happiness: using the new positive psychology to realize your potential for lasting fulfillment. New York: Free Press, xiv, 321.

[ref63] Simonsen I.-E. Rundmo T. (2020). The role of school identification and self-efficacy in school satisfaction among Norwegian high-school students. Soc. Psychol. Educ. 23, 1565–1586. doi: 10.1007/s11218-020-09595-7

[ref64] Snyder C. R. (2000). Handbook of hope: theory, measures, and applications. San Diego: Academic Press.

[ref65] Stanley M. S. (1996). School uniforms and safety. Educ. Urban Soc. 28, 424–435. doi: 10.1177/0013124596028004003

[ref66] Tajfel H. Turner J. (2001). “An integrative theory of intergroup conflict. Intergroup relations: essential readings” in Key readings in social psychology, Hove: Psychology Press 94–109.

[ref67] Tho N. D. Quynh Thu N. N. (2026). Students’ psychological capital and learning performance: a multilevel study of the roles of instructors’ mindfulness and forgiveness. Int. J. Manag. Educ. 24:101319. doi: 10.1016/j.ijme.2025.101319

[ref68] Turner J. C. Hogg M. A. Oakes P. J. Reicher S. D. Wetherell M. S. (1987). Rediscovering the social group: a self-categorization theory. Oxford: Basil Blackwell.

[ref69] Verkuyten M. (2021). Group identity and ingroup bias: the social identity approach. Hum. Dev. 65, 311–324. doi: 10.1159/000519089

[ref70] Wolfe M. J. (2024). School uniforms that hurt: an Australian perspective on gendered mattering. Aust. Educ. Res. 51, 909–927. doi: 10.1007/s13384-023-00682-0

[ref71] Woo J. M. Tam C. L. Bonn G. B. Tagg B. (2020). Student, teacher, and school counselor perceptions of national school uniforms in Malaysia. Front. Psychol. 11:1871. doi: 10.3389/fpsyg.2020.01871, 32849098 PMC7399159

[ref72] Woof V. G. Hames C. Speer S. Cohen D. L. (2021). A qualitative exploration of the unique barriers, challenges and experiences encountered by undergraduate psychology students with mental health problems. Stud. High. Educ. 46, 750–762. doi: 10.1080/03075079.2019.1652809

[ref73] Worley J. T. Smith A. L. (2026). Positive peer relationships, social identity, and adaptive sport motivation in youth athletes. Psychol. Sport Exerc. 82:102996. doi: 10.1016/j.psychsport.2025.102996, 40939807

[ref74] You J. W. (2016). The relationship among college students’ psychological capital, learning empowerment, and engagement. Learn. Individ. Differ. 49, 17–24. doi: 10.1016/j.lindif.2016.05.001

